# Crystal structure of full-length *Zika virus* NS5 protein reveals a conformation similar to *Japanese encephalitis virus* NS5

**DOI:** 10.1107/S2053230X17001601

**Published:** 2017-02-21

**Authors:** Anup K. Upadhyay, Matthew Cyr, Kenton Longenecker, Rakesh Tripathi, Chaohong Sun, Dale J. Kempf

**Affiliations:** a AbbVie Inc., 1 North Waukegan Road, North Chicago, IL 60064, USA

**Keywords:** *Zika virus*, NS5, nonstructural protein 5

## Abstract

The rapid spread of the recent *Zika virus* (ZIKV) epidemic across various countries in the American continent poses a major health hazard for the unborn fetuses of pregnant women. To date, there is no effective medical intervention. The nonstructural protein 5 of *Zika virus* (ZIKV-NS5) is critical for ZIKV replication through the 5′-RNA capping and RNA polymerase activities present in its N-terminal methyltransferase (MTase) and C-terminal RNA-dependent RNA polymerase (RdRp) domains, respectively. The crystal structure of the full-length ZIKV-NS5 protein has been determined at 3.05 Å resolution from a crystal belonging to space group *P*2_1_2_1_2 and containing two protein molecules in the asymmetric unit. The structure is similar to that reported for the NS5 protein from *Japanese encephalitis virus* and suggests opportunities for structure-based drug design targeting either its MTase or RdRp domain.

## Abstract 2

The recent epidemic of *Zika virus* (ZIKV) has created a major global health concern. Here, the crystal structure of the full-length ZIKV NS5 protein at 3.05 Å resolution is presented to facilitate the structure-based design of antiviral agents against ZIKV.

## 1. Introduction   

The spread of *Zika virus* (ZIKV) through more than 35 countries in the American continents over the past year has created a major public health emergency (Lessler *et al.*, 2016 ▸; Hajra *et al.*, 2016 ▸). ZIKV is spread primarily through *Aedes albopictus* and *A. aegypti* mosquito vectors, but can also be sexually transmitted (Petersen *et al.*, 2016 ▸; Weaver *et al.*, 2016 ▸). Acute infection with ZIKV is often subclinical or, if symptomatic, typically associated with mild symptoms characteristic of acute viral infection. However, an increased risk of severe neurological disease, in particular Guillain–Barre syndrome, has been associated with ZIKV infection (Cao-Lormeau *et al.*, 2016 ▸). Furthermore, ZIKV infection causes decreased male fertility in mice (Govero *et al.*, 2016 ▸). Most concerning, however, is the microcephaly associated with viral replication in human fetal brain tissue following perinatal transmission in pregnant women (Garcez *et al.*, 2016 ▸; Broutet *et al.*, 2016 ▸; Carteaux *et al.*, 2016 ▸).

ZIKV is a member of the *Flaviviridae* virus family, genus *Flavivirus*. It is closely related to other flaviviruses such as *Dengue virus* (DENV1–4), *Yellow fever virus* (YFV), *Japanese encephalitis virus* (JEV) and *West Nile virus* (WNV) (Petersen *et al.*, 2016 ▸; Weaver *et al.*, 2016 ▸). *Flaviviridae* are single positive-stranded RNA viruses that undergo cytoplasmic replication in host cells by a replication complex containing nonstructural protein 5 (NS5) as the RNA polymerase. Somewhat more distantly related are members of the genus *Hepacivirus*, which include the important human pathogen *Hepatitis C virus* (HCV; Giangaspero *et al.*, 2008 ▸). Nucleoside and non-nucleoside inhibitor classes targeting the HCV NS5B RNA polymerase have been shown to be effective in combination with other inhibitor classes to profoundly block viral replication and cure HCV infection (Zhang *et al.*, 2016 ▸).

**Figure 1:**
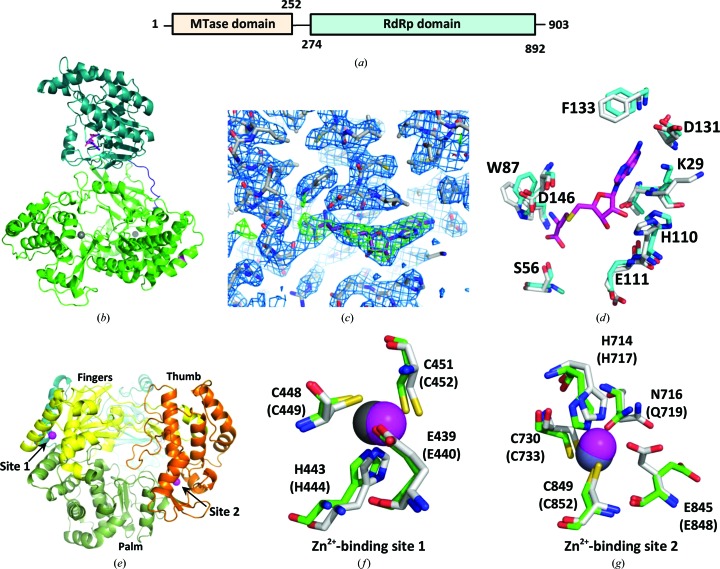
(*a*) Domain architecture of ZIKV-NS5(1–903) protein. (*b*) X-ray structure of the full-length ZIKV-NS5 protein, where residues 6–887 are observed in the structure. The MTase domain is shown in teal and the RdRp domain is shown in green. The bound cofactor (SAH) in the MTase domain is highlighted in magenta, and the two Zn^2+^ ions in the RdRp domain are shown in gray. The linker region connecting the MTase and RdRp domains is shown in blue. (*c*) Representative section of the electron-density map for the protein with the bound cofactor (SAH) in the MTase domain. The weighted 2*F*_o_ − *F*_c_ map (blue) is contoured at 1σ and a difference OMIT map (green) for the SAH is contoured at 3σ. (*d*) The conserved residues involved in SAH (magenta) binding in the NS5 MTase domain are shown for the JEV-NS5 (gray; PDB entry 4k6m) and ZIKV-NS5 (cyan; PDB entry 5tfr) structures. (*e*) ZIKV-NS5 pol oriented with a ‘right-handed’ view of the palm (olive), thumb (orange) and fingers (yellow) domains, where the Mtase (blue) domain extends into the page. (*f*, *g*) Coordination environments of the two Zn^2+^-binding sites. ZIKV-NS5 residues are shown in green and the two Zn^2+^ ions are shown in gray. JEV-NS5 residues are shown in gray and the corresponding Zn^2+^ ions are shown in magenta.

Like other flaviviruses, ZIKV contains a linear genome encoding, from the 5′-end, three structural proteins (capsid, membrane and envelope) and seven nonstructural proteins (NS1, NS2a, NS2b, NS3, NS4a, NS4b and NS5) (Cunha *et al.*, 2016 ▸). The NS5 proteins from flaviviruses are unique among RNA viruses for having a fused domain architecture comprised of an N-terminal RNA methyltransferase (MTase) domain and a C-terminal RNA-dependent RNA polymerase (RdRp) domain (Fig. 1 ▸ *a*). The N-terminal MTase domain is responsible for 5′ capping and thereby stabilizes the viral RNA genome, while the C-terminal RdRp domain is critical for viral RNA replication (Decroly *et al.*, 2011 ▸; Zhao, Soh, Lim *et al.*, 2015 ▸; Lu & Gong, 2013 ▸). Both of these two domains are potential therapeutic targets for developing antiviral drugs. X-ray crystal structures of the NS5 proteins from various flaviviruses have been solved and used for the structure-based drug design of novel antivirals (Lim *et al.*, 2011 ▸; Benmansour *et al.*, 2016 ▸; Malet *et al.*, 2008 ▸). Here, we report the first X-ray crystal structure of the full-length NS5 polymerase from ZIKV in complex with *S*-adenosylhomocysteine (SAH), with the hope of aiding efforts towards the identification of antiviral drugs to help mitigate current and future ZIKV epidemics.

## 2. Materials and methods   

### 2.1. Protein expression and purification   

The cDNA sequence encoding the full-length *Zika virus* strain MR766 RNA-dependent RNA polymerase NS5 protein ZIKV-NS5(1–903) (NCBI Reference Sequence YP_009227205.1) was synthetically generated (GenScript) and cloned into pET-28b expression vector as a N-terminally His_6_-tagged protein with an internal thrombin cleavage site for removal of the His_6_ tag after purification. The recombinant protein was expressed in *Escherichia coli* BL21-CodonPlus (DE3)-RIPL strain. The cells were grown in Luria Broth (LB) medium containing 50 mg l^−1^ kanamycin and 34 mg l^−1^ chloramphenicol at 37°C until the cell density (OD_600_) reached 0.4; the temperature was then lowered to 16°C. Protein expression was induced by adding 0.5 m*M* IPTG to the medium and growing the cells overnight at 16°C. The cell pellet was resuspended in lysis buffer (20 m*M* Tris–HCl pH 7.5, 500 m*M* NaCl, 10 m*M* imidazole, 10% glycerol, 1 m*M* MgCl_2_, 1 m*M* TCEP) containing EDTA-free protease-inhibitor cocktail set V (Calbiochem, catalog No. 539137) and 50 units of turbonuclease (Accelagen, catalog No. N0103M) per gram of cell paste. The resuspended cells were lysed by passage through an emulsifier at 83 MPa pressure. The cell lysate was clarified by centrifugation at 25 000*g* for 1 h and the supernatant was loaded onto a nickel-affinity column pre-equilibrated in the lysis buffer. The bound proteins were eluted by running a linear gradient of 10–500 m*M* imidazole over 20 column volumes and collected as 5 ml fractions. The fractions containing the desired ZIKV-NS5(1–903) protein were pooled together and digested with thrombin (Sigma, catalog No. T4648) while dialyzing overnight at 4°C in 20 m*M* Tris–HCl pH 7.5 buffer containing 500 m*M* NaCl, 10% glycerol and 1 m*M* TCEP. The cleaved His_6_ tag and thrombin were removed by passing the thrombin-treated protein through 5 ml Ni Sepharose 6 Fast Flow resin (GE Healthcare) and 1 ml *p*-aminobenzamidine-agarose beads (Sigma, catalog No. A-7155), respectively. The cleaved protein was further purified by size-exclusion chromatography on a Superdex S200 gel-filtration column using 20 m*M* Tris–HCl pH 7.5, 500 m*M* NaCl, 10% glycerol, 1 m*M* TCEP as the final protein buffer. The purity of the purified protein was assessed by polyacrylamide gel electrophoresis (SDS–PAGE). The protein concentration was determined by UV absorbance at 280 nm with a NanoDrop 2000 spectrophotometer (Thermo Scientific) using a theoretical molar extinction coefficient of 224 890 *M* ^−1^ cm^−1^.

### 2.2. Crystallization   

The purified protein was concentrated to 12 mg ml^−1^ and set up for crystallization at 17°C. The final optimized crystals were obtained from hanging-drop conditions using 0.1 *M* sodium cacodylate (pH 5.9–6.9) and trisodium citrate (0.6–1.2 *M*) as well solutions. For data collection, the crystals were cryoprotected by dipping them into a solution containing 80% well solution and 20%(*v*/*v*) glycerol and then flash-cooled in liquid nitrogen. Crystallization information is given in Table 1 ▸.

### 2.3. Data collection and processing   

Multiple crystals were screened to identify a crystal with reasonable diffraction. The best data set was collected to 3.05 Å resolution under gaseous nitrogen (100 K) on the IMCA-CAT beamline (17-ID) at the Advanced Photon Source at Argonne National Laboratory, Argonne, Illinois, USA using X-rays at a wavelength of 1.0 Å. Diffraction intensities were processed using *autoPROC* (Vonrhein *et al.*, 2011 ▸). Data-collection and refinement statistics are shown in Table 2 ▸.

### 2.4. Structure solution and refinement   

The structure was solved by molecular replacement with the coordinates of JEV-NS5 (PDB entry 4k6m; Lu & Gong, 2013 ▸) using *Phaser* within the *CCP*4 program suite (Winn *et al.*, 2011 ▸; Table 3 ▸). The model was rebuilt using *Coot* (Emsley *et al.*, 2010 ▸) and refined against structure factors using *autoBUSTER* (Smart *et al.*, 2012 ▸). The two molecules in the asymmetric unit are very similar, with the protein structure built as a continuous chain for residues 6–887. Both exhibit additional densities for the bound cofactor *S*-adenosylhomocysteine (SAH) in each MTase domain and two zinc ions bound on each polymerase domain. With significant anisotropy in the diffraction data (anisotropic ratio = 1.3), treatment with the *StarAniso* algorithm (Global Phasing Ltd) aided iterative refinement of the model. Figures were prepared using *PyMOL* (Schrödinger).

## 3. Results   

Full-length *Zika virus* NS5 protein [ZIKV-NS5(1–903)] was purified from an *E. coli*-based expression system and the purified protein was crystallized at 17°C. The structure was solved at 3.05 Å resolution by molecular replacement using the published JEV-NS5 structure (PDB entry 4k6m) as a search model (Table 3 ▸; Lu & Gong, 2013 ▸). Residues 6–887 of the protein sequence were modeled as a continuous chain into the observed electron-density map (Fig. 1 ▸ *b*). The N-terminal MTase domain showed additional density for the bound cofactor SAH that was copurified from the bacterial expression host (Fig. 1 ▸ *c*). SAH is the demethylated form of *S*-adenosylmethionine (SAM), which is the major methyl donor for eukaryotic and prokaryotic methyltransferase enzymes. Co-purifications of SAH- or SAM-bound methyltransferase enzymes from bacterial expression hosts are quite common and have been reported previously for the DENV and ZIKV NS5 proteins (Coloma *et al.*, 2016 ▸; Egloff *et al.*, 2002 ▸; Lim *et al.*, 2011 ▸). The residues surrounding the SAH-binding sites are conserved and adopt similar conformations among the related structures (Fig. 1 ▸ *d*).

The C-terminal RdRp domain of the ZIKV-NS5 protein adopts the thumb, palm and fingers motifs (Fig. 1 ▸ *e*) characteristic of polymerase folds, as reported for other flavivirus NS5 proteins and the distantly related HCV-NS5B protein (Lu & Gong, 2013 ▸; Sesmero & Thorpe, 2015 ▸). There are two zinc-binding sites in the RdRp domain. Site 1 (Fig. 1 ▸ *f*) is conserved in JEV-NS5 and binds a Zn^2+^ ion by coordinating through residues Glu439, His443, Cys448 and Cys451. Site 2 (Fig. 1 ▸ *g*) utilizes residues His714, Cys730 and Cys849 as in JEV, but orients the side chain of the fourth residue (Glu845) away and uses Asn716 instead of Gln as the fifth residue to coordinate the zinc. Site 2 has been suggested to play a regulatory role in modulating movement between the thumb and the palm of the RdRp domain (Lu & Gong, 2013 ▸; Yap *et al.*, 2007 ▸); however, the functional consequence of this subtle structural difference is not yet well understood.

**Figure 2:**
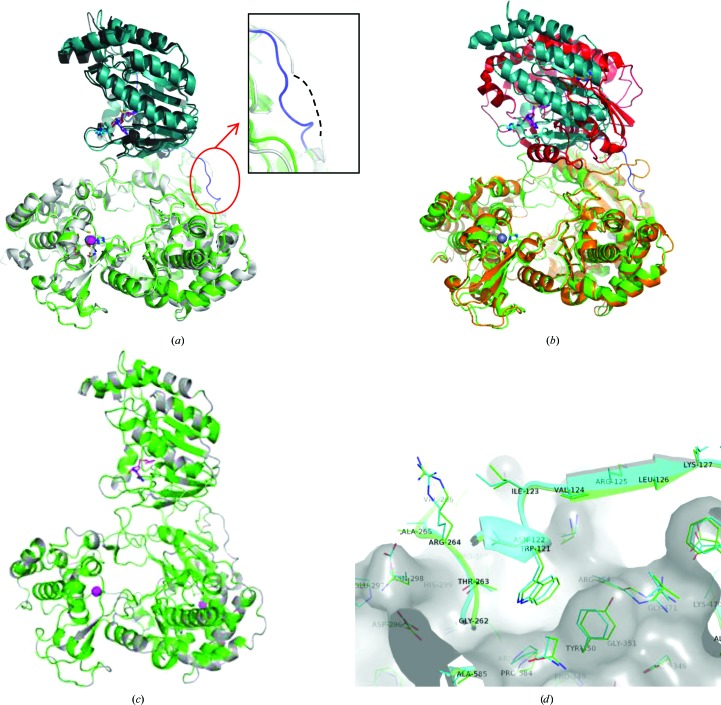
Comparison of NS5 structures from ZIKV (PDB entry 5tfr), JEV (PDB entry 4k6m) and DENV3 (PDB entry 5ccv). (*a*) Overlay of the full-length ZIKV-NS5 structure with the JEV-NS5 structure (PDB entry 4k6m). The JEV-NS5 protein is shown in gray. The ZIKV-NS5 protein is shown in teal (MTase domain) and green (RdRp domain). The inset shows the linker regions of the two proteins (blue, ZIKV-NS5; gray, JEV-NS5). (*b*) Overlay of the DENV3-NS5 structure with the ZIKV-NS5 protein. The DENV3-NS5 protein is shown in red (MTase domain) and orange (RdRp domain). The ZIKV-NS5 protein is shown as in (*a*). (*c*) Sequence homology between the ZIKV-NS5 and JEV-NS5 proteins is shown by highlighting the conserved residues between the two proteins in green on the ZIKV-NS5 structure. (*d*) Structural overlay of the ZIKV-NS5 and JEV-NS5 proteins at the interface between the MTase and RdRp domains with conserved residues involved in the interaction.

The MTase domain of the ZIKV-NS5 protein folds onto the RdRp domain in a manner similar to that observed in the JEV-NS5 structure (Lu & Gong, 2013 ▸). Unlike the JEV-NS5 structure, the linker region (residues 266–275) connecting the MTase and RdRp domains has continuous electron density to enable modeling of all of the residues. The relative spatial orientation of the two domains in the ZIKV-NS5 protein is similar (0.94 Å r.m.s.d.) to that of the JEV-NS5 protein (Fig. 2 ▸ *a*) but distinctly different from the DENV3-NS5 protein (Fig. 2 ▸ *b*; PDB entry 5ccv; Klema *et al.*, 2016 ▸). Consistent with the 67% sequence identity (Fig. 2 ▸ *c*) and the observed structural similarities (Fig. 2 ▸ *a*) between the ZIKV-NS5 and JEV-NS5 proteins, the residues present at the interface of the two domains are highly conserved between these two proteins and also adopt similar conformations (Fig. 2 ▸ *d*). The RdRp and MTase domains in flaviviral NS5 proteins are reported to cross-talk with each other to synergize their RNA polymerase and 5′-capping functions (Zhao, Soh, Chan *et al.*, 2015 ▸; Zhao, Soh, Zheng *et al.*, 2015 ▸). The linker region and the interacting residues at the interface of the two domains are shown to play critical roles in regulating the enzymatic functions of flaviviral NS5 proteins and impact viral replication (Zhao, Soh, Zheng *et al.*, 2015 ▸). Compounds that destabilize this inter-domain interaction may therefore function as potential allosteric inhibitors for flaviviral NS5 enzymes.

## 4. Discussion   

ZIKV is an example of several pathogenic human viruses that disproportionally affect people living in poverty with limited access to healthcare (Hotez *et al.*, 2009 ▸). The expansion of *Chikungunya virus* and *Dengue virus* (Furuya-Kanamori *et al.*, 2016 ▸) and the recent west African outbreak of *Ebola virus* (WHO Ebola Response Team, 2016 ▸) illustrate the need for cooperation between the public and private sectors to develop new antiviral drugs to aid in responding to future epidemics. The availability of detailed structural information from X-ray crystallographic studies on viral proteins can assist the discovery of new agents alongside high-throughput screening, repurposing of known antivirals and other drug-discovery techniques (Eyer *et al.*, 2016 ▸; Elfiky, 2016 ▸; Julander *et al.*, 2016 ▸). In particular, iterative structure-based drug design based on co-crystallography of enzyme–inhibitor structures of viral polymerases has been especially fruitful in the optimization of agents for other viral diseases (Lim *et al.*, 2011 ▸; Sesmero & Thorpe, 2015 ▸). Here, we report the first X-ray crystal structure of the full-length NS5 protein from ZIKV, containing both MTase and RdRp domains. We anticipate that this structure may enable the discovery and structure-based optimization of new agents to treat or possibly prevent ZIKV infection.

The current ZIKV-NS5 structure compares very closely with the published JEV-NS5 structure (Fig. 2 ▸ *a*), suggesting a similar behavior in functional properties and inter-domain dynamics. While the domain positioning of ZIKV-NS5 contrasts with those reported for DENV-NS5 structures, the individual domains in these proteins share similar structural features, as expected from the high sequence homology among the flaviviruses. The diffraction data in the current study offer insight into the larger structural features, but the interpretation of detailed atomic positions remains limited. Future studies should allow structural characterization at higher resolution with more details of local features. Some strategies that are likely to improve diffraction quality include truncations of the currently disordered N-terminal (amino acids 1–5) and C-terminal (amino acids 888–903) residues of the protein to promote different crystal packing. This may also reduce the anisotropic diffraction pattern that we have observed in the current crystal form (see §2.4). Alternatively, crystallization of the individual MTase and RdRp domains, as evident from the recently published high-resolution X-ray structure of the ZIKV-NS5 MTase domain (Coloma *et al.*, 2016 ▸), may also aid in obtaining high-resolution structural information on the individual domains. In addition, further characterizations through biophysical and biochemical functional assays are needed to understand the structure–function relationships in the context of inhibitor screening.

In conclusion, the global reach of the ZIKV epidemic emphasizes the need for multiple approaches to stem its expansion and prevent its devastating sequelae in unborn children. Although the development of effective vaccines may have the greatest impact, new antivirals that block ZIKV replication may also be important for treating or preventing ZIKV-associated morbidities. Here, we report the first X-ray crystal structure of the full-length NS5 protein from ZIKV, which may be useful in the discovery and optimization of such antivirals.

## Supplementary Material

PDB reference: full-length *Zika virus* NS5 protein, 5tfr

## Figures and Tables

**Table 1:** Crystallization conditions

Method	Hanging-drop vapour diffusion
Plate type	24-well plates
Temperature (K)	290
Protein concentration (mg ml^−1^)	12
Buffer composition of protein solution	20 m*M* Tris–HCl pH 7.5, 500 m*M* NaCl, 10% glycerol, 1 m*M* TCEP
Composition of reservoir solution	0.1 *M* sodium cacodylate (pH 5.9–6.9), trisodium citrate (0.6–1.2 *M*)
Volume and ratio of drop	1:1
Volume of reservoir (ml)	0.8

**Table 2:** Data collection and processing Values in parentheses are for the outer shell.

Diffraction source	17-ID IMCA-CAT, APS
Wavelength (Å)	1.00
Temperature (K)	100
Detector	Dectris PILATUS 6M
Crystal-to-detector distance (mm)	450
Rotation range per image (°)	0.3
Total rotation range (°)	180
Exposure time per image (s)	0.1
Space group	*P*2_1_2_1_2
*a*, *b*, *c* (Å)	136, 196, 95
α, β, γ (°)	90, 90, 90
Mosaicity (°)	0.1–0.2
Resolution range (Å)	100–3.05 (3.10–3.05)
Total No. of reflections	330316 (15565)
No. of unique reflections	49315 (2433)
Completeness (%)	99.99 (99.6)
Multiplicity	6.7 (6.4)
〈*I*/σ(*I*)〉	8 (2.1)
*R*_meas_	23 (99)
Overall *B* factor from Wilson plot (Å^2^)	68

**Table 3:** Structure solution and refinement Values in parentheses are for the outer shell.

Resolution range (Å)	45–3.05 (3.13–3.05)
Completeness (%)	99.9 (99.6)
No. of reflections, working set	49224 (3447)
No. of reflections, test set	2471 (154)
Final *R*_cryst_	0.188 (0.262)
Final *R*_free_	0.237 (0.353)
No. of non-H atoms	
Protein	14188
Ion	4
Ligand	52
Water	229
Total	14473
R.m.s. deviations	
Bonds (Å)	0.010
Angles (°)	1.14
Average *B* factors (Å^2^)	
Protein	61
Ion	71
Ligand	70
Water	38
Ramachandran plot	
Most favored (%)	97.3
Allowed (%)	2.5
